# Microglial TYROBP/DAP12 in Alzheimer’s disease: *Transduction of physiological and pathological signals across TREM2*

**DOI:** 10.1186/s13024-022-00552-w

**Published:** 2022-08-24

**Authors:** Jean-Vianney Haure-Mirande, Mickael Audrain, Michelle E. Ehrlich, Sam Gandy

**Affiliations:** 1 Department of Neurology, Icahn School of Medicine at Mount Sinai, New York, NY 10029 USA; 2 Department of Pediatrics, Icahn School of Medicine at Mount Sinai, New York, NY 10029 USA; 3 Department of Genetics and Genomic Sciences, Icahn School of Medicine at Mount Sinai, New York, NY 10029 USA; 4 Department of Psychiatry and the NIA-Designated Mount Sinai Alzheimer’s Disease Research Center, Icahn School of Medicine at Mount Sinai, New York, NY 10029 USA; 5 James J Peters VA Medical Center, New York, Bronx NY 10468 USA

**Keywords:** Tyrobp/Dap12, Trem2, ApoE, miR-155, Disease-Associated Microglia (DAM), Sensome, Complement C1q, Alzheimer, Amyloid, Tau

## Abstract

TYROBP (also known as DAP12 or KARAP) is a transmembrane adaptor protein initially described as a receptor-activating subunit component of natural killer (NK) cells. TYROBP is expressed in numerous cell types, including peripheral blood monocytes, macrophages, dendritic cells, and osteoclasts, but a key point of recent interest is related to the critical role played by TYROBP in the function of many receptors expressed on the plasma membrane of microglia. TYROBP is the downstream adaptor and putative signaling partner for several receptors implicated in Alzheimer’s disease (AD), including SIRP1β, CD33, CR3, and TREM2. TYROBP has received much of its current notoriety because of its importance in brain homeostasis by signal transduction across those receptors. In this review, we provide an overview of evidence indicating that the biology of TYROBP extends beyond its interaction with these four ligand-binding ectodomain-intramembranous domain molecules. In addition to reviewing the structure and localization of TYROBP, we discuss our recent progress using mouse models of either cerebral amyloidosis or tauopathy that were engineered to be TYROBP-deficient or TYROBP-overexpressing. Remarkably, constitutively TYROBP-deficient mice provided a model of genetic resilience to either of the defining proteinopathies of AD. Learning behavior and synaptic electrophysiological function were preserved at normal physiological levels even in the face of robust cerebral amyloidosis (in *APP/PSEN1*;*Tyrobp*^*−/−*^ mice) or tauopathy (in *MAPT*^*P301S*^;*Tyrobp*^*−/−*^ mice). A fundamental underpinning of the functional synaptic dysfunction associated with each proteotype was an accumulation of complement C1q. TYROBP deficiency prevented C1q accumulation associated with either proteinopathy. Based on these data, we speculate that TYROBP plays a key role in the microglial sensome and the emergence of the disease-associated microglia (DAM) phenotype. TYROBP may also play a key role in the loss of markers of synaptic integrity (e.g., synaptophysin-like immunoreactivity) that has long been held to be the feature of human AD molecular neuropathology that most closely correlates with concurrent clinical cognitive function.

## Background

The proteinopathies and neuroimmune/neuroinflammatory features of Alzheimer’s disease (AD) neuropathology underpin a major proportion of late-life cognitive failure. The AD-related proteinopathies include cerebral interstitial, cerebrovascular, and meningovascular deposition of amyloid-β (Aβ) peptides composed of 38-43 amino acid species that form unstructured oligomers and highly structured fibrils [1]. The full range of biological activities associated with these Aβ species remains to be fully elucidated. Recent evidence implicates some direct cytotoxic mechanisms wherein Aβ oligomers have been proposed to be more bioactive while Aβ fibrils have been proposed to be more inert. Evidence has recently developed independently from multiple labs and experimental systems that implicate interactions of Aβ oligomers with cell-surface prion protein (PrP), isotype 5 of the metabotropic glutamate receptor and/or intracellular protein kinases, e.g., PERK, Fyn, and cdk5 [2–4].

In addition to the possibility of direct neurotoxicity due to various Aβ-derived species, neuroinflammatory pathways can be driven not only by Aβ-derived oligomeric and fibrillar species but also by aggregates of microtubule-associated protein TAU (MAPT) that can assemble to form intracellular neurofibrillary tangles in neurons and/or glia. AD neuropathology includes not only amyloidosis and tauopathy, as many brains harboring AD neuropathology are also reported to contain Lewy body α-synuclein pathology [5, 6]. This property is apparent even among brains of subjects harboring autosomal dominant familial AD pathogenic mutations in *APP, PSEN1,* or *PSEN2* [7]. Each of these proteinopathies can influence each other and trigger and/or exacerbate immune-inflammatory events that almost always involve microglia, the central nervous system resident macrophages [8].

Microglia perform sentinel functions within the brain, regulating homeostasis and rapidly reacting to damage, infections and/or debris by adapting their phenotypes and phagocytic capacity. Despite their roles as “first responder” cells, the microglia arriving at the sites of AD-related proteinopathies can soon see their clean-up capacities outstripped, which, associated with the production of proinflammatory cytokines, can contribute to disease progression and neurodegeneration [9].

In addition to their phagocytosis-related duties, microglia support neurons by modulating neuronal activity [10] and by pruning synapses [11–13] especially during brain development [14, 15]. However, this process can be improperly activated during aging [16] and disease [17, 18]. Recent evidence demonstrates that oligomeric Aβ, in the company of C3, C1q, and apolipoprotein E (APOE), can drive pathological pruning that may play a role in how Aβ oligomers cause loss of synapses and lead to cognitive decline [19]. Another mechanism of Aβ oligomer–induced synapse loss may involve planar cell polarity (PCP) signaling components [20]. PCP signaling regulates synapse formation and maintenance during brain development [21], and recent evidence shows that binding of Aβ oligomers to PCP components is required for Aβ oligomer–induced synapse loss. Blockage of PCP signaling using monoclonal antibodies decreases microglial activation, protects synapses from Aβ oligomer–induced loss, and preserves cognitive function [20]. Other evidence points to a type 1 interferon-related toxicity that degrades synaptic function and/or integrity [17]. However, the relationship(s) among oligomeric Aβ-C3-C1q-APOE*ε4-related microglial synaptic pruning and type 1 interferon-related synaptic toxicity remain(s) to be clarified.

Research in the past few years has advanced our understanding of the molecular events underpinning the involvement of microglia in the etiology of neurodegenerative diseases. Genome-wide association studies (GWAS) have identified many AD-associated variants associated with microglia [22–24], and recent analyses revealed specific transcriptomic signatures for quiescent or “homeostatic” microglia when compared with disease-associated microglia (DAM) [25, 26]. Additionally, new approaches using human induced pluripotent stem cell-derived microglia [27], single-cell and single-nucleus RNA sequencing have contributed to our understanding of microglia in AD and AD-associated immune-inflammatory events. These observations support a formulation wherein microglia are not mere “spectators” during neurodegeneration but rather function as “initiators” and/or “active participants” in the progression of AD and neurodegenerative diseases.

GWAS-related approaches to human AD have identified candidate risk loci related to microglia in late-onset AD (LOAD). Two of the most studied are *APOE* and *TREM2* (for Triggering Receptor Expressed on Myeloid Cells 2). TYROBP (for tyrosine kinase binding protein; also known as *DAP12* for DNAX activating protein-12 and as *KARAP* for killer cell-activating receptor-associated protein) is a transmembrane polypeptide that acts as an adaptor for several receptors on microglia and other myeloid cells. Interestingly, TYROBP forms a necessary bridge between TREM2 at the microglial cell surface and APOE transcription within microglia. In 2013, Zhang et al. performed a multiscale gene network analysis combining neuropathology, whole-genome genotyping, and RNA-sequencing analyses, and identified *TYROBP* as a key network hub gene and driver in sporadic LOAD patients [28]. Concurrently, Hickman et al. (2013) identified a family of 100 transcripts highly enriched in microglia during a sensing activity and showed that *TYROBP* was a hub of this “sensome” [29]. Recently, our lab has confirmed in two mouse models of AD-related proteinopathies the critical events that can occur when *Tyrobp* gene expression is not optimized [30–33].

One explanation for this gene dose optimization requirement has been presented for outcomes when the inhibition of the tyrosine-protein kinase ABL has been targeted to alleviate neurodegenerative diseases [34]. In this study, authors showed that broad inhibition of multiple tyrosine-protein kinases using low doses of inhibitors was more effective at reducing Aβ and tau toxicities and alleviating neurodegenerative pathologies than was ABL inhibition alone using more selective inhibitors [34]. Since TYROBP is a tyrosine kinase substrate phosphoprotein, this gene dose optimization phenomenon may be relevant not only for tyrosine kinases, but also for phospho-tyrosine-status-sensitive physiological effector substrates such as TYROBP.

Golde and colleagues have described another example of the requirement for empirical gene dose optimization studies [35]. In this example, they designed a protocol aimed at exploring the effect of the expression of the anti-inflammatory cytokine, interleukin-10 (IL-10), on Aβ pathology in the brains of two mouse models of amyloid pathology (TgCRND8 and Tg2576). In contrast to several reports demonstrating that proinflammatory cytokines promote amyloid pathology [36–38], IL-10 expression increased Aβ accumulation, decreased synaptic markers, and impaired learning behavior in the *APP* mice. Interestingly, transcriptomic analyses revealed increased APOE mRNA levels in mice expressing IL-10 that were associated with increased APOE protein levels within the plaque-associated insoluble cellular fraction. These observations suggest that proinflammatory and anti-inflammatory signals can negatively affect amyloid pathology and cognition and demonstrate the complex interplay between innate immunity and proteostasis in neurodegenerative diseases, an interaction that Golde and colleagues have named “immunoproteostasis” [35]. Given the complex role that TYROBP plays in integrating the interrelationships linking environmental sensing, signal transduction, and phagocytosis, the term “immunoproteostasis” might well apply to TYROBP gene expression manipulation in our hands in much the same way that IL-10 manipulation yielded unexpected results in the hands of Golde and colleagues [35].

In this review, we introduce the structure and signaling of TYROBP and present an overview of its roles in the brain. We discuss the most notable advances on the role of TYROBP in AD pathogenesis. Specifically, we summarize our previous reports investigating the possibility that modulation of TYROBP levels or activity might play a role in cognitive resilience. In particular, we outline our observations demonstrating that TYROBP deficiency in mouse models of AD-related proteinopathies sustains normal learning behavior and electrophysiological functions even in the face of robust amyloidosis or tauopathy and that TYROBP pathways may act, at least in part, via complement-mediated and/or ERK-related pathways. Because of these data and others, we suggest that the microglial sensome is not the exclusive province of TREM2.

##  TYROBP structure and signaling

**Fig. 1:**
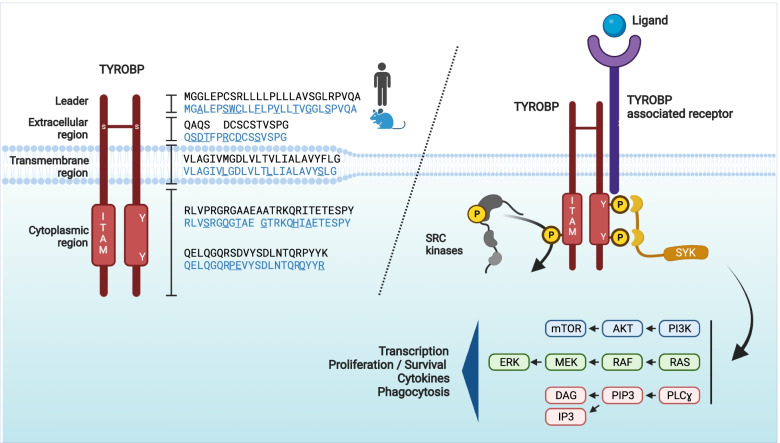
TYROBP structure and signaling pathway. Left panel: Mouse and human TYROBP: the TYROBP protein consists of a 27-aa leader peptide, a small 14-aa (16-aa in mouse, in blue) extracellular region, a 24-aa transmembrane segment, and a 48-aa (49-aa in mouse) cytoplasmic domain. Because of two cysteine residues (Cys33/35) in its extracellular region, TYROBP forms a disulfide-bonded homodimeric complex. TYROBP contains an immunoreceptor tyrosine-based activation motif (ITAM) in its cytoplasmic region. Right panel: Following ligand-binding by a TYROBP-associated receptor, the ITAM motif can be phosphorylated on its two conserved tyrosine residues by SRC kinases and induce the intracellular recruitment and activation of the spleen tyrosine kinase (SYK). Upon SYK recruitment, several downstream effector molecules such as phosphatidylinositol 3-kinase (PI3K), the small GTPase RAS, or the phospholipase Cγ (PLCγ) are mobilized, resulting in activation of transcription, proliferation, release of cytokines, and phagocytosis. Abbreviations: DAG, diacylglycerol; ERK, extracellular signal-regulated kinase; IP3, inositol trisphosphate; ITAM, immunoreceptor tyrosine-based activation motif; MEK, mitogen-activated protein kinase kinase; PI3K, phosphatidylinositol 3-kinase; PIP3, phosphatidylinositol-3,4,5-trisphosphate; PLCγ, phospholipase Cγ; SYK, spleen tyrosine kinase; TYROBP, tyrosine kinase binding protein; Y, Tyrosine

Initially described in NK cells as KARAP [39–41], TYROBP is expressed in peripheral tissues in multiple lymphoid and myeloid cell subtypes, including osteoclasts. In the brain, TYROBP is mostly expressed in microglia but also in dendritic cells. The human *TYROBP* gene is located on chromosome 19 (7 in mouse) and encodes a 113 (114 in mouse) amino acid (aa) type I transmembrane adapter protein (Fig. 1). The TYROBP protein consists of a 27-aa leader peptide, a small 14-aa (16-aa in mouse) extracellular region, a 24-aa transmembrane segment, and a 48-aa (49-aa in mouse) cytoplasmic domain. Because of two cysteine residues (Cys33/35) in its extracellular region, TYROBP forms a disulfide-bond-crosslinked homodimeric protein. TYROBP contains an immunoreceptor tyrosine-based activation motif (ITAM) in its cytoplasmic region. This ITAM motif is the only signaling domain identified for TYROBP and can be phosphorylated by SRC on one or both of two highly conserved tyrosine residues. The characterization of the differential protein-protein interactions between the spleen tyrosine kinase (SYK) and mono- vs. di-phosphorylated ITAM tyrosyl residues of TYROBP is an area of ongoing interest (https://ftp.wwpdb.org/pub/pdb/validation_reports/q5/7q5w/7q5w_full_validation.pdf.gz). The tyrosyl phosphorylated form of TYROBP [42] induces the intracellular recruitment and activation of protein kinases, including the spleen tyrosine kinase (SYK) in myeloid cells and SYK and ZAP70 in NK cells. Upon SYK recruitment, several downstream effector molecules, including phosphatidylinositol 3-kinase (PI3K), the small GTPase RAS, and/or the phospholipase Cγ (PLCγ) are mobilized.

As is the case for many immune system receptors, TYROBP is part of a multi-subunit protein complex in which the recognition of a specific ligand and the downstream intracellular signaling domain are the product of distinct subunits. Multi-protein complexes involving TYROBP and various receptor ectodomains-intramembranous domains are formed through a complex electrostatic network involving the hydrophilic intramembranous domain residues [43, 44]. Ligation of TYROBP-associated receptors to TYROBP provides docking sites for various ligands that can initiate intracellular signals through TYROBP and its ITAM motif.

Most of these pathways trigger microglial activation with increased release of cytokines (such as IL-1, IL-6, or TNFɑ), cytoskeletal rearrangement, and phagocytosis. However, it has been reported that TYROBP can also inhibit phagocytosis [45–48]. To explain how some TYROBP-associated receptors promote activation signals while others amplify cellular inhibition, Turnbull and Colonna (2007) proposed an avidity-based model [49]. Thus, while a high-avidity interaction between a ligand and a TYROBP-associated receptor would induce a complete phosphorylation of TYROBP in the ITAM motif and the recruitment of SYK, a low-avidity interaction would induce an incomplete phosphorylation of TYROBP and the activation of the inhibitory phosphatase SH2-domain-containing protein tyrosine phosphatase 1 (SHP-1, encoded by *PTPN6*), leading to the induction of inhibitory signaling [49].

## TYROBP receptors in the brain

Many receptors have been identified that act by furnishing ligand-binding ectodomains and short transmembrane domains to the TYROBP adaptor [49–51], and some of these receptors are expressed in human and rodent brains and are known to be associated with AD **(**Table 1**).** For example, the signal regulatory protein-β1 (SIRPβ1) is a microglial receptor associated with TYROBP [52, 53]. SIRPβ1 expression is increased in the brains of patients and animal models of AD pathology, and this molecule was previously linked to the phagocytosis and clearance of debris and Aβ aggregates [55]. However, the natural ligand for SIRPβ is unknown.

**Table 1:** Microglial and myeloid cell-surface receptors associated with TYROBP

Receptors	Gene	Species	Ligands	Ref.	Implication for disease	Ref.
SIRPß1	*SIRPB1*	Human, mouse	Unknown	[52–54]	Increased expression in AD	[55]
TREM1	*TREM1*	Human, mouse	PGLYRP1, HMGB1, HSP70, extracellular actin	[56–59]	Septic shock, pneumonia, bacterial infectious diseases	[60–62]
TREM2	*TREM2*	Human, mouse	ApoE, Aβ oligomers, phospholipids, lipopolysaccharide	[63–66]	Nasu-Hakola disease, early-onset AD, FTD	[67–69]
TREM3	*Trem3*	Mouse	Unknown	[70]	Unknown	
TREM5	*CD300LB*	Human, mouse	Unknown	[71]	Unknown	
MDL1 (CLEC5a)	*MDL1*	Human, mouse	Fucose and mannose, membrane glycans, hemagglutinin protein of influenza viruses	[72–74]	Chronic obstructive pulmonary disease, viral infections	[74, 75]
SIGLEC-14	*SIGLEC14*	Human	Glycans	[76, 77]	Unknown	
SIGLEC-15	*SIGLEC15*	Human, mouse	Glycans, tumor-associated glycan structure, CD44	[78, 79]	Osteopetrosis	[80]
SIGLEC-16	*SIGLEC16*	Human, mouse	Glycans	[81]	Unknown	
SIGLEC-H	*SiglecH*	Mouse	Unknown	[82, 83]	Unknown	
pDC-TREM	*pDC-Trem*	Mouse	Unknown	[84]	Unknown	
PILRB	*PILRB*	Human, mouse	Sialylated O-linked sugars	[85–88]	Unknown	
mCD33 (SIGLEC-3)*	*Siglec3*	Mouse	Glycans	[82]	Polymorphisms of human CD33 linked to AD	[89–91]
IREM2	*CD300E*	Human, mouse	Unknown	[92, 93]	Unknown	
MAIR-II (LMIR2, CLM-4, DIgR1)	*Cd300C2*	Mouse	Unknown	[94–96]	Unknown	

*AD* Alzheimer’s disease, *ApoE* Apolipoprotein E, *Aβ* Amyloid-β, *CD300LB* CD300 -Like Family Member B, *CLEC5* C-Type Lectin Domain Containing 5A, *Siglec* Sialic Acid-Binding Immunoglobulin-Type Lectins, *FTD* Frontotemporal dementia, *mCD33* Mouse CD33, *MAIR-II* Myeloid-associated immunoglobulin-like receptor, *pDC-TREM* Plasmacytoid Dendritic Cells-Triggering Receptor Expressed On Myeloid Cells, *SIGLEC-14* Sialic Acid Binding Ig-Like Lectin 14, *SIGLEC-15* Sialic Acid Binding Ig-Like Lectin 15, *SIGLEC-16* Sialic Acid Binding Ig-Like Lectin 16, *SIGLEC-H* Sialic Acid Binding Ig-Like Lectin H, *SIRPβ1* Signal Regulatory Protein-Β1, *TREM1* Triggering Receptor Expressed On Myeloid Cells 1, *TREM2* Triggering Receptor Expressed On Myeloid Cells 2, *TREM3* Triggering Receptor Expressed On Myeloid Cells 3, *TREM5* Triggering Receptor Expressed On Myeloid Cells 5, *TYROBP* Tyrosine Kinase Binding Protein, *mCD33: mouse CD33, based on the sequence similarity in the transmembrane domain with that of mouse SIGLEC-H

The TYROBP receptor that has received the most attention in the AD field is TREM2 because of the discovery in 2013 that patients harboring the *TREM2*^*R47H*^ variant are at increased relative risk for developing AD similar to that associated with the APOE*ε4 allele [97, 98]. Heretofore, APOE*ε4 had been anticipated to be the most potent genetic factor that was likely to exist for enhancing the relative risk for AD. Additional rare variants of *TREM2* have been discovered (e.g., R62H, T96K, D87N), and, for the most part, these variants are shown or are predicted to confer a loss of function of TREM2 by decreasing the ligand-binding capacity or cell surface expression. The TREM2 extracellular domain is the receptor part of the protein that binds many ligands, including phospholipids, Aβ oligomers, DNA, and bacterial lipopolysaccharide. Interestingly, several apolipoproteins, including APOE, bind to TREM2 and this binding is abolished or reduced in the presence of *TREM2* disease variants [63, 64, 99]. Because of the absence of signaling motifs in the TREM2 intracellular domain, full-length TREM2 almost certainly exerts its actions via TYROBP. The long extracellular ectodomain can also be cleaved by proteases such as ADAM10, ADAM17, or meprin ß, liberating soluble TREM2 (sTREM2). sTREM2 has been shown to be biologically active in enhancing the production of inflammatory cytokines and microglia survival [100], binding oligomeric Aβ [101], and blocking Aβ oligomerization and fibrillization [102]. Several studies also reported that sTREM2 was positively correlated with cerebrospinal fluid (CSF) levels of phosphorylated TAU [103, 104]. Although oligomeric Aβ can induce sTREM2 shedding and viral infections such as HIV increased levels of sTREM2 in the CSF, conditions that modulate sTREM2 shedding from full-length TREM2 are not fully understood [102, 105] and may occur to block the activity of full-length TREM2 and its downstream signaling. Also, by binding similar ligands, sTREM2 and full-length TREM2 may share some biological functions that do not involve interaction with TYROBP. More experiments aimed at deciphering the precise role of sTREM2 and full-length TREM2/TYROBP complex in AD are needed.

Other TREM receptors present on the myeloid cells, such as TREM1, TREM3, and TREM5, can associate with TYROBP. Of note, while a low level of TREM3 is detected in mouse microglia, TREM3 in humans is present as a pseudogene [56, 70, 106]. Although like most TYROBP-associated receptors, their ligands are unknown and their roles in brain biology are not fully understood, TREM1 has been implicated in septic shock and bacterial infectious diseases [60–62] demonstrating the role of TYROBP-associated receptors in the immune response against bacterial infections.

CLEC5a (or MDL1) is a member of the C-type lectin/C-type lectin-like domain superfamily and has been shown to interact with TYROBP [72]. Ligands of CLEC5a include the fucose and mannose sugars of the dengue virus and the hemagglutinin protein of influenza viruses [73, 74]. These data further support the role of the TYROBP pathway in the immune response against pathogens.

Members of the sialic acid-binding immunoglobulin-type lectins (Siglec) proteins comprise another class of receptors that associates with TYROBP. Siglecs are mainly expressed in immune cells and recognize glycans in sialic acid. The Siglec family contains 15 members in humans and 8 members in mice. Siglecs are rapidly evolving genes and are partially conserved across species. The majority of the Siglecs contain intracellular Immunoreceptor Tyrosine-based Inhibitory Motifs (ITIMs) and recruit the inhibitory phosphatase SHP-1, leading to the induction of inhibitory intracellular signaling. Still, some Siglecs have a positive amino acid charge (lysine or arginine residues) in the transmembrane domain and can associate with TYROBP and transduce signals through ITAM motifs (Siglecs-14, − 15, − 16) [76–78]. However, human RNAseq data from purified cell types suggest a relatively low expression level in microglia, with Siglec-14 being expressed at a higher level than Siglec-15 and -16 (http://www.brainrnaseq.org). The precise roles of these Siglecs in the brain and microglia remain to be investigated. A mouse-specific Siglec (Siglec-H) was the first Siglec to be reported to associate with TYROBP [82]. Siglec-H is expressed in dendritic cells, macrophages, and microglia [83, 107]. Although the mechanism is not fully understood, the activation of TYROBP signaling by Siglec-H inhibits the production of type I interferon (IFN) [108]. Further elucidation of this link involving TYROBP and Siglec-H may converge with type I IFN signaling in causing or exacerbating synaptic dysfunction and degeneration in AD [17].

CD33 (also known as Siglec-3) was identified as a top-ranked AD risk factor by GWAS [89]. Elevated levels of CD33 protein were reported in AD brain and were associated with amyloid pathology and disease progression [90, 109]. Moreover, de Jager and colleagues reported a positive correlation of CD33 protein level with that of TREM2 in humans. The mouse and human CD33 are substantially different proteins in expression patterns and ligand recognition [110]. Notably, unlike human CD33 which contains two ITIM motifs and associates with SHP-1[111–113], mouse CD33 lacks one of the ITIM motifs and is suggested to interact with, and signal through, TYROBP [82]. In *APP/PSEN1* mice, the absence of CD33 decreased the brain levels of Aβ42 and amyloid plaque burden [91], suggesting a negative role of CD33 in amyloid uptake by microglia. Of note, we observed a downregulation of *Cd33* and *Trem2* in the brains of *APP/PSEN1* mice in the absence of TYROBP [33]. Although mouse and human CD33 studies may not be directly comparable due to sequence dissimilarities and signaling, our results dovetail with those of de Jager and colleagues and suggest regulation of *Cd33* and *Trem2* expression by TYROBP.

## TYROBP in Alzheimer’s disease and mouse models of cerebral amyloidosis or tauopathy

Since 2013 and the identification by Zhang and colleagues of *TYROBP* as a key network driver in sporadic LOAD [28], several reports have confirmed the crucial involvement of TYROBP in AD pathology and progression. TYROBP expression is increased in AD patients and AD mouse models [28, 114], and rare *TYROBP* missense coding variants have been identified in patients with familial early-onset AD [115]. Moreover, as discussed above, TYROBP acts as a downstream adaptor for microglial receptors known to interact with Aβ. Finally, deletion and premature termination, loss-of-function mutations in the *TYROBP* gene are associated with Nasu-Hakola disease, a rare recessively-inherited disease associated with early-onset dementia [116].

**Fig. 2:**
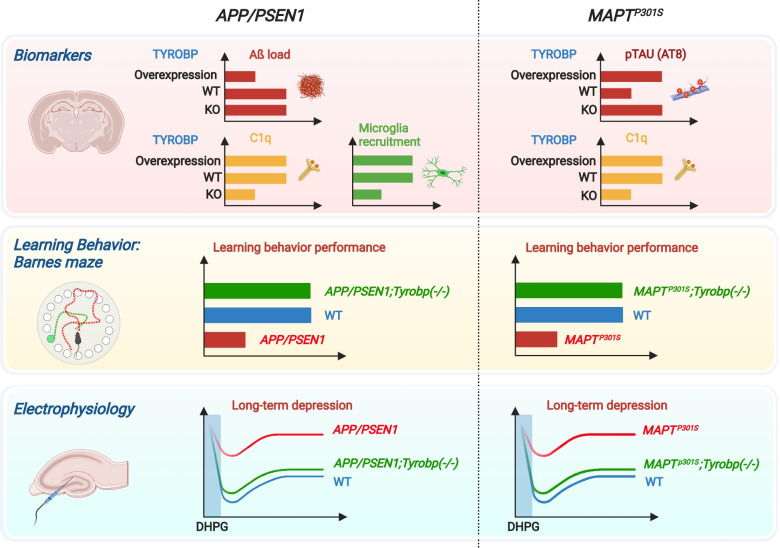
TYROBP influences AD pathology in standard AD-related mouse models of cerebral amyloidosis or tauopathy. In *APP/PSEN1* mice*,* absence of TYROBP decreased microglial recruitment around Aβ deposits. A*PP/PSEN1* mice overexpressing TYROBP showed decreased Aβ load. In *MAPT*^*P301S*^ (PS19) mice, both absence and overexpression of TYROBP increased the stoichiometry of phosphorylation of TAU. While observations of these TAU phenotypic effects are usually associated with worsening clinical phenotype, absence of *Tyrobp* decreased the expression of C1q, the initiating protein in the classical complement cascade, and improved learning behavior and synaptic function in both amyloidosis and tauopathy mouse models. Abbreviations: TYROBP, tyrosine kinase binding protein; C1q, Complement component 1q; WT, Wildtype; KO, Knockout; DHPG, Dihydroxyphenylglycine

**Fig. 3:**
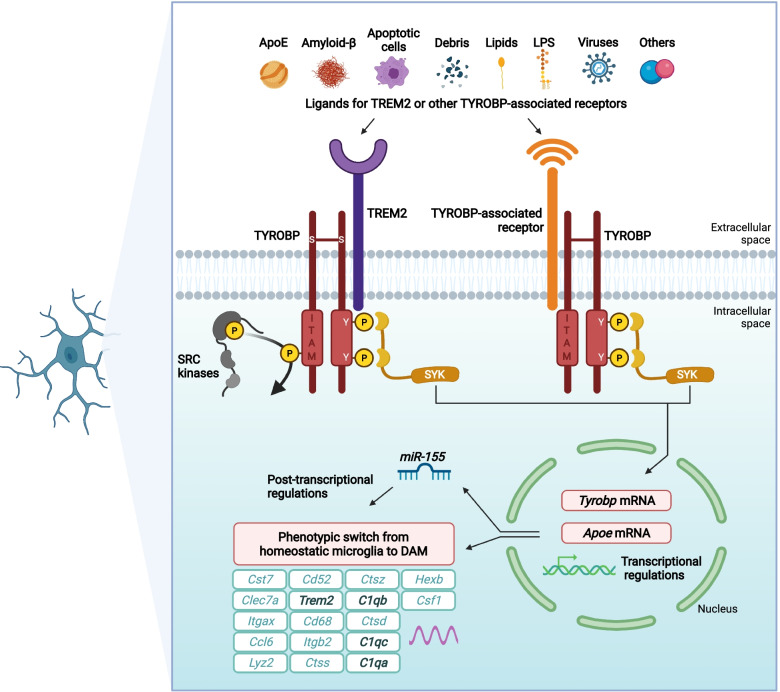
Probable ligand-induced TYROBP signaling in recruited microglia. Ligand-induced TYROBP signaling is initiated by apolipoprotein E, Aβ, debris, or other currently unidentified ligands at sensing receptors and leads to phosphorylation of the tyrosine residues in the cytoplasmic ITAM of TYROBP by SRC kinases and the recruitment of SYK. In turn, SYK signaling can increase transcription of *Tyrobp* and *ApoE*. This series of events forms the basis for the phenotypic switch from homeostatic microglia to DAM and may be in part post-transcriptionally regulated by *miR-155*. We speculate that in mice lacking *Trem2*, recruited microglia cause other receptor ectodomains to acquire sensing capability either because these receptors always signal by complexing with TYROBP, or because signals across other complexes are induced as a compensatory mechanism due to the absence of *Trem2*. Abbreviations: APOE, apolipoprotein E; C1q, complement protein C1q; Ccl6, C-C motif chemokine ligand 6; Cd52, CD52 molecule; Cd68, CD68 molecule; Clec7a, C-Type lectin domain containing 7A; Csf1, colony stimulating factor 1; Cst7, cystatin F; Ctsd, cathepsin D; Ctss, cathepsin S; Ctsz, cathepsin Z; DAM, disease-associated microglia; Hexb, hexosaminidase subunit beta; ITAM, immunoreceptor tyrosine-based activation motif; Itgax, integrin subunit alpha X; Itgb2, integrin subunit beta 2; Lyz2, lysozyme 2; miR-155, microRNA-155; SYK, spleen tyrosine kinase; TREM2, triggering receptor expressed on myeloid cells-2; TYROBP, tyrosine kinase binding protein; Y, tyrosine. Adapted and reprinted with permission from Wiley Publishing, Hoboken, NJ

Our lab characterized the in vivo consequences of TYROBP deficiency in standard AD-related mouse models of cerebral amyloidosis or tauopathy. Although in our hands the absence of TYROBP did not affect the total number of microglia in the cortex and hippocampus of WT or AD-related mouse models as compared to mice with WT level of TYROBP, we showed that a constitutive deficiency of TYROBP in the *APP/PSEN1* mice, a mouse model of the cerebral amyloidosis of AD, reduced the clustering of microglia around Aβ plaques. Interestingly, *APP/PSEN1* mice deficient for TYROBP maintained normal learning behavior assays and electrophysiology recordings at both 4 and 8 months of age (Fig. 2). In these same TYROBP-deficient, *APP/PSEN* mice, we observed a dramatic reduction of most of the *Tyrobp*-driven complement subnetwork, as previously observed from human brain RNA sequencing [28, 32, 33]. We also observed repression of the induction of genes involved in the switch from homeostatic microglia to DAM, including *Trem2*, *Clec7a*, and *Cst7* (Fig. 3). In addition, the absence of TYROBP in the *APP/PSEN1* mice decreased the expression of *Irf8*, a transcription factor implicated in microglia development, regulation of proinflammatory cytokine expression, and recently involved in microglial migration and microglial spread of Aβ [117–119].

When crossed with the PS19 tauopathy mouse model (*MAPT*^*P301S*^), mice with a *Tyrobp* deletion showed evidence for an increased stoichiometry of phosphorylation of TAU as well as enhanced transneuronal diffusion of TAU. These observations were confirmed in *Tyrobp*^−/−^ mice injected with AAV-TAU [31]. Despite these phenotypic effects usually associated with worsening clinical phenotype, *MAPT*^*P301S*^ mice deficient for TYROBP showed improved learning behavior and synaptic function, similar to what we observed in *APP/PSEN1* mice (Fig. 2) [31, 32]. Interestingly, one common molecular property shared between *APP/PSEN1;Tyrobp*^−/−^ and *MAPT*^*P301S*^*;Tyrobp*^−/−^ mice was the reduction of the level of complement protein C1q, suggesting that deletion of TYROBP drives beneficial effects in learning behavior and synaptic functions in part via decreased C1q levels [31, 32] (Fig. 2).

Recently, we interrogated the consequences of TYROBP upregulation in microglia. We developed a novel transgenic mouse overexpressing TYROBP and crossed it with *APP/PSEN1* and *PS19* mice [30]. Similar to TYROBP deficiency in the setting of our studies, TYROBP overexpression did not affect the total number of microglia in the brain. We reported a decrease of brain amyloid burden in the *APP/PSEN1* mice overexpressing TYROBP and an increase of TAU phosphorylation stoichiometry in the *MAPT*^*P301S*^ mice overexpressing TYROBP, surprisingly similar to results obtained in the *MAPT*^*P301S*^*;Tyrobp*^−/−^ mice [31]. These data confirm the crucial role of microglia and TYROBP in both amyloidosis and tauopathy progression (Fig. 2) and the complex interplay between innate immunity and proteostasis in neurodegenerative diseases [37].

## TYROBP and the complement system

The complement system was initially described in the periphery and consists of multiple proteins in a cascade that enhances the immune system to react to microbes, pathogens, or other damaging agents. The system performs analogous functions in the brain with the clearance of damaged cells, unwanted cellular materials, and even synapses [11, 12]. Synaptic pruning is a physiological process described initially during the development to eliminate weaker synapses to the advantage of more robust and more active synapses. Still, recent studies show that the process can be inappropriately activated during aging [16] and AD [17, 18]. C1q, the initiating protein in the classical complement cascade, is mainly produced by microglia in the brain [120] and is also part of the complement subnetwork driven by TYROBP [28]. Both TYROBP and C1q were recently identified as key predictors of gastric cancers [121], supporting a strong link between these two proteins even beyond AD. C1q is upregulated in amyloidosis and tauopathy mouse models [32, 114, 122] and, as described above, our team observed that a deficiency of TYROBP induces a dramatic reduction of C1q in both *APP/PSEN1* and *MAPT*^*P301S*^ mice (Fig. 2) [31, 32]. This decrease is associated with the maintenance of normal learning behavior and electrophysiological properties, supporting suggestions from others that C1q is a target of interest in the treatment of neurodegenerative diseases [11, 18]. TYROBP and C1q transcripts are upregulated in close proximity to amyloid plaques in the human AD brain, while TREM2 transcripts are not closely approximated to plaques [114]. This seems to confirm that microglia sense plaques and respond by activating the complement cascade through the release of C1q, apparently recognizing the plaque as a “foreign” conformation despite its origin from a proteolytically processed “self” holoprotein, human APP. The tagging of neurons by C1q may be a trigger leading to neuronal dystrophy, tau phosphorylation, and neurodegeneration.

## TYROBP: A hub for phagocytosis signaling pathways?

As previously mentioned, TYROBP interacts closely with receptors involved in phagocytosis, and a substantial body of evidence indicates that several key receptors involved in amyloid clearance or compaction are closely linked to TYROBP. In preparation for phagocytosis, a respiratory burst generates reactive oxygen species and involves an interaction between TYROBP and complement receptor 3 (CR3). CR3 is a heterodimer consisting of the integrins CD11b and CD18, and is an important TYROBP-related receptor in microglia. While the direct binding between TYROBP and CD11b/CD18 is still debated, several reports have demonstrated that TYROBP is required for signaling downstream of CR3. Syk and the ITAM of TYROBP are, for example, required for integrin signaling in neutrophils and macrophages [123, 124]. Additionally, CD11b acts through TYROBP in microglia-mediated neuronal apoptosis during development [125]. Notably, in the current discussion, blocking CR3 signaling -- if initiated before the accumulation of AD-related cerebral amyloidosis -- can protect synapses in the brains of mouse models from that component of AD pathology [126]. Our reports suggest a direct regulation of CR3 by TYROBP which may mediate, at least in part, the beneficial effects of TYROBP deletion on learning behavior and synaptic functions. Thus, we showed that *APP/PSEN1* mice deficient for TYROBP displayed decreased expression of *Itgam* encoding for CD11b (RNAseq, adj *p*-value = 0.02) [32]. On the other hand, *MAPT*^*P301S*^ mice overexpressing TYROBP displayed a trend toward increased *Itgam* (qPCR, *p*-value = 0.12) (Audrain et al., unpublished data).

Recently, Huang et al. (2021) highlighted the role of TAM receptors AXL and MER in amyloid plaque construction by microglia in *APP/PSEN1* mice [127]. They showed that AXL and MER detect and react to amyloid plaques and that deletion of either of these genes blocks phagocytosis and “organization” of the plaques by microglia [127]. We observed a similar phenotype in the *APP/PSEN1* mice deficient for TYROBP [32, 33]. *Axl* transcripts were decreased (RNAseq, adj p-value = 0.27, p-value = 0.004) in 8 month-old *APP/PSEN1;Tyrobp*^−/−^ vs. *APP/PSEN1* [32], but were increased in 4 month-old *APP/PSEN1* mice overexpressing TYROBP vs. *APP/PSEN1* (qPCR, p-value = 0.0092) [30]. Further investigation is required to understand the complex interrelationships that underpin these various receptors and adaptors and their actions in the orchestration of phagocytosis.

## TYROBP, sensome, and microglial switching

Microglia constantly monitor the brain environment, and many alterations of brain homeostasis can induce a microglial response. Several transcriptomic studies describe distinct signatures for microglia that define their states from healthy to disease-associated. These transcriptomic profiles are essential to an elucidation of which genes and proteins are required to trigger specific responses depending on the alterations faced by microglia and the surrounding cells with which they communicate. Using single-cell sequencing in *5xFAD* mice, Keren-Shaul et al. (2017) showed a unique subtype of microglia, referred to as Disease-Associated Microglia (DAM), localized near the amyloid plaques [26]. Using *Trem2*^−/−^ mice, they identified two sequential but distinct stages in the switch to a DAM phenotype. The first step is *Trem2*-independent and requires the activation of genes that include *Tyrobp* and *Apoe*. The second step was found to be *Trem2*-dependent and associated with phagocytosis. A report published by Krasemann et al. (2017) identified a similar signature in microglia acquiring a neurodegeneration-associated phenotype (MGnD) in models of amyotrophic lateral sclerosis (ALS), multiple sclerosis (MS), and AD, and pointed to microRNA-155 (*miR-155*) as an effector of the MGnD phenotype [25]. This study and a report from Butovsky et al. [128] showed that ablation of *Trem2*, *Apoe,* or *miR-155* locks microglia into a homeostatic state blocking the formation of MGnD. Of note, our recent reports placed *miR-155* at the intersection of a multiplex of AD pathogenic components involving innate immunity, viral response, synaptic physiology, and pro-amyloidogenic pathways [129]. Although similar, the study by Krasemann et al. (2017) contrasts with Keren-Shaul et al. (2017), in which *Apoe* upregulation appeared to be independent of *Trem2*. More recently, Chen et al. (2020) used a combination of spatial transcriptomics and in situ sequencing to avoid one of the main drawbacks of these types of analyses, i.e., averaging the transcriptomes between microglia which are and are not recruited around the amyloid plaques [114]. They investigated the transcriptional changes occurring in a 100 μm diameter around the amyloid plaques of *APP*^*NL-G-F*^ mice and defined a plaque-induced gene (PIG) network of 57 genes in which *Trem2*, *Tyrobp*, *Apoe*, and other complement-related genes were among the upregulated genes. When performed in human AD brain slices, both *Tyrobp* and *Apoe* transcripts were confirmed as enriched, but, unexpectedly, *Trem2* was not among the human PIGs. As stated above, our lab showed that the absence of *Tyrobp* in *APP/PSEN1;Tyrobp*^−/−^ mice represses the induction of many genes involved in this DAM switch, including *Trem2*, complement (*C1qa*, *C1qb*, *C1qc*, and *Itgax*), *Clec7a* and *Cst7* (Fig. 3) [32]. Furthermore, we recently provided evidence that concurrent upregulation in microglia of both *Tyrobp* and *Apoe* is interconnected during microglial sensing of amyloid deposits and that these events take place independent of *Trem2* but are dependent on *Tyrobp* (Fig. 3) [30]. The damage-associated signatures described in all DAM, MGnD, and PIG microglia suggest that microglial transition from a homeostatic to a disease-associated state is choreographed by multiple components and involves TREM2, TYROBP, and APOE. Our data confirmed the two stages described by Keren-Shaul et al. (2017), in which the first stage would eventually correspond to a sensing of the amyloid plaques by the microglia and where both *Tyrobp* and *Apoe* are upregulated. The first stage is independent of *Trem2* and would be followed by a second stage that is dependent on *Trem2* and mostly associated with phagocytosis.

## Link between Nasu-Hakola and Alzheimer's diseases?

Nasu–Hakola disease, also known as polycystic lipomembranous osteodysplasia with sclerosing leukoencephalopathy (PLOSL), is characterized by multiple bone cysts and progressive presenile dementia. This rare disease was first described in 1973 [130, 131], and most patients have been identified in Japan and Finland. PLOSL is caused by autosomal recessive deletion or premature termination loss of function mutations in either *TREM2* or *TYROBP* [116]. This appears to distinguish PLOSL mutations in *TREM2* or *TYROBP* from the missense mutations that associate TYROBP with AD [115]. TYROBP and TREM2 are expressed in most innate immune and myeloid cells, including osteoclasts. By differentiating peripheral blood mononuclear cells isolated from Nasu-Hakola patients, Paloneva et al. (2000) showed that loss of function mutations in both *TREM2* and *TYROBP* induced a delayed and sub-optimal differentiation of osteoclasts associated with bone resorption [116]. The neurological stage of the disease is clinically similar to a frontal lobe syndrome and frontotemporal dementia (FTD), and FTD can be difficult to distinguish clinically from AD. Although *TREM2* and *TYROBP* mutations are causative for Nasu-Hakola disease and increase the risk of AD, the link between these two dementias is still unclear. Aβ deposits have been observed in the cortex of a 39-year-old woman with the *TREM2* Q33X mutation and Nasu-Hakola disease [132]. However, Satoh et al. (2018) studied the expression of Aβ in five Nasu-Hakola cases and concluded that amyloid plaques were almost undetectable in these brains [133]. Interestingly, other studies revealed that TREM2/TYROBP interacts closely with the colony-stimulating factor 1 receptor (CSF1R) [123, 134]. Strikingly, there is a CSF1R mutation that causes white matter disease with dementia termed hereditary diffuse leukoencephalopathy with spheroids (HDLS) [135]. Thus, both Nasu-Hakola and HDLS could involve similar or identical microglial pathway(s). Further studies in large populations will be essential to evaluate whether associations exist between TYROBP and other dementias.

## Concluding remarks

Microglial biology and pathobiology have converged to create a highly active research area in AD and other neurodegenerative diseases. The rate of publication of microglia-related papers per year has tripled over the past decade. However, the range of microglial actions and their abilities to worsen or attenuate AD makes microglia-oriented interventions still uncertain. Specifically, whether microglia should be inhibited or activated, and when in the course of clinical or preclinical AD microglia can be targeted effectively, are all key pieces of information that currently remain unknown [136]. TREM2 has provided a valuable object lesson since many investigators proposed that upregulating TREM2 would be beneficial since AD-associated variants specify loss of function [137]. Interestingly, a recent antisense oligonucleotide (ASO) strategy showed that acute reduction of *Trem2* in *APP/PSEN1* mice decreases amyloid deposition by increasing microglial phagocytosis [138]. This confirms our recent report showing that, even in the absence of *Trem2,* microglia can still sense the presence of amyloid plaques [30].

Furthermore, many additional microglial receptors interacting directly and indirectly with TYROBP are involved in AD pathogenesis. TYROBP is one of the two main ITAM-containing adaptors that mediate signaling by heterologous receptors [139]. This suggests that various extracellular stimuli — acting through TREM2, Toll-like receptors, and other receptors — can lead to activation of intracellular signaling pathways, suggesting that altering the activity of a single microglial signaling pathway may not be sufficient for full activation.

The association of TYROBP with sporadic and early-onset AD and its role as a key regulator for many AD-associated functions of microglia, including phagocytosis, complement activation, synaptic pruning, and a switch from homeostatic to DAM state, suggest that the modulation of TYROBP level or activity may represent a therapeutic opportunity in AD. Although the potential “druggability” of microglial receptors and adapters such as TYROBP may present challenges due to the interactions of each with numerous ligands and signaling pathways, the recent discovery of CNS-penetrating small molecules like sobetirome and Sob-AM2, two thyromimetic agents capable of targeting TREM2, may ultimately lead to a viable therapeutic approach to selective manipulation of individual microglial events [140]. Overall, a better understanding of TYROBP and its many interactors and pathways will improve our understanding of the role(s) played by microglia in the etiology and pathogenesis of human neurodegenerative diseases.

## Data Availability

Not applicable.

## Abbreviations

AD: Alzheimer’s disease
ALS: Amyotrophic Lateral Sclerosis
ApoE: Apolipoprotein E
ASO: Antisense Oligonucleotide
Aβ: Amyloid-β
CD300LB: CD300-Like Family Member B
CLEC5: C-Type Lectin Domain Containing 5A
CSF: Cerebrospinal Fluid
Siglec: Sialic Acid-Binding Immunoglobulin-Type Lectins
CSF1R: Colony-Stimulating Factor 1 Receptor
DAM: Disease-Associated Microglia
DAP12: DNAX Activating Protein-12
ERK: Extracellular Signal-Regulated Protein Kinase
FTD: Frontotemporal Dementia
GWAS: Genome-Wide Association Studies
HDLS: Hereditary Diffuse Leukoencephalopathy with Spheroids
IFN: Type I Interferon
IL-12/23: Interleukin-12/23
ITAM: Immunoreceptor Tyrosine-Based Activation Motif
ITIM: Immunoreceptor Tyrosine-Based Inhibitory Motifs
KARAP: Killer Cell-Activating Receptor-Associated Protein
LOAD: Late-Onset AD
MAPT: Microtubule-Associated Protein TAU
mCD33: Mouse CD33
MGnD: Microglial Neurodegenerative Phenotype
miR-155: microRna-155
MS: Multiple Sclerosis
MAIR-II: Myeloid-associated immunoglobulin-like receptor
NK Cells: Natural Killer Cells
PCP: Planar Cell Polarity
PI3K: Phosphatidylinositol 3-Kinase
pDC-TREM: Plasmacytoid Dendritic Cell-Triggering Receptor Expressed On Myeloid Cells
PIG: Plaque-Induced Genes
Plcγ: Phospholipase Cγ
PLOSL: Polycystic Lipomembranous Osteodysplasia with Sclerosing Leukoencephalopathy
PrP: Prion Protein
SHP-1: SH2-Domain-Containing Protein Tyrosine Phosphatase 1
SIGLEC-14: Sialic Acid Binding Ig-Like Lectin 14
SIGLEC-15: Sialic Acid Binding Ig-Like Lectin 15
SIGLEC-16: Sialic Acid Binding Ig-Like Lectin 16
SIGLEC-H: Sialic Acid Binding Ig-Like Lectin H
SIRPβ1: Signal Regulatory Protein-Β1
sTREM2: Soluble TREM2
SYK: Spleen Tyrosine Kinase
TAM: Tyro3, AXL, and MER
TREM1: Triggering Receptor Expressed On Myeloid Cells 1
TREM2: Triggering Receptor Expressed On Myeloid Cells 2
TREM3: Triggering Receptor Expressed On Myeloid Cells 3
TREM5: Triggering Receptor Expressed On Myeloid Cells 5
TYROBP: Tyrosine Kinase Binding Protein

## References

[1] Glabe CG (2008). Structural classification of toxic amyloid oligomers. J Biol Chem.

[2] Um JW, Kaufman AC, Kostylev M, Heiss JK, Stagi M, Takahashi H, Kerrisk ME, Vortmeyer A, Wisniewski T, Koleske AJ (2013). Metabotropic glutamate receptor 5 is a coreceptor for Alzheimer abeta oligomer bound to cellular prion protein. Neuron.

[3] Raka F, Di Sebastiano AR, Kulhawy SC, Ribeiro FM, Godin CM, Caetano FA, Angers S, Ferguson SSG (2015). Ca2+/Calmodulin-dependent protein Kinase II interacts with group I Metabotropic Glutamate and facilitates Receptor Endocytosis and ERK1/2 signaling: role of β-Amyloid. Molecular brain.

[4] Joshi G, Chi Y, Huang Z, Wang Y (2014). Abeta-induced Golgi fragmentation in Alzheimer's disease enhances Abeta production. Proc Natl Acad Sci USA.

[5] Hansen L, Salmon D, Galasko D, Masliah E, Katzman R, DeTeresa R, Thal L, Pay MM, Hofstetter R, Klauber M (1990). The Lewy body variant of Alzheimer's disease: a clinical and pathologic entity. Neurology.

[6] Hamilton RL (2000). Lewy bodies in Alzheimer's disease: a neuropathological review of 145 cases using alpha-synuclein immunohistochemistry. Brain Pathol.

[7] Twohig D, Nielsen HM (2019). alpha-synuclein in the pathophysiology of Alzheimer's disease. Mol Neurodegener.

[8] Lloyd GM, Dhillon JS, Gorion KM, Riffe C, Fromholt SE, Xia Y, Giasson BI, Borchelt DR (2021). Collusion of alpha-Synuclein and Abeta aggravating co-morbidities in a novel prion-type mouse model. Mol Neurodegener.

[9] Hickman SE, Allison EK, El Khoury J (2008). Microglial dysfunction and defective beta-amyloid clearance pathways in aging Alzheimer's disease mice. J Neurosci.

[10] Badimon A, Strasburger HJ, Ayata P, Chen X, Nair A, Ikegami A, Hwang P, Chan AT, Graves SM, Uweru JO (2020). Negative feedback control of neuronal activity by microglia. Nature.

[11] Stevens B, Allen NJ, Vazquez LE, Howell GR, Christopherson KS, Nouri N, Micheva KD, Mehalow AK, Huberman AD, Stafford B (2007). The classical complement cascade mediates CNS synapse elimination. Cell.

[12] Schafer DP, Lehrman EK, Kautzman AG, Koyama R, Mardinly AR, Yamasaki R, Ransohoff RM, Greenberg ME, Barres BA, Stevens B (2012). Microglia sculpt postnatal neural circuits in an activity and complement-dependent manner. Neuron.

[13] Schafer DP, Stevens B (2015). Microglia Function in Central Nervous System Development and Plasticity. Cold Spring Harb Perspect Biol.

[14] Paolicelli RC, Bolasco G, Pagani F, Maggi L, Scianni M, Panzanelli P, Giustetto M, Ferreira TA, Guiducci E, Dumas L (2011). Synaptic pruning by microglia is necessary for normal brain development. Science.

[15] Tremblay ME, Stevens B, Sierra A, Wake H, Bessis A, Nimmerjahn A (2011). The role of microglia in the healthy brain. J Neurosci.

[16] Stephan AH, Madison DV, Mateos JM, Fraser DA, Lovelett EA, Coutellier L, Kim L, Tsai HH, Huang EJ, Rowitch DH (2013). A dramatic increase of C1q protein in the CNS during normal aging. J Neurosci.

[17] Roy ER, Wang B, Wan YW, Chiu G, Cole A, Yin Z, Propson NE, Xu Y, Jankowsky JL, Liu Z (2020). Type I interferon response drives neuroinflammation and synapse loss in Alzheimer disease. J Clin Invest.

[18] Hong S, Beja-Glasser VF, Nfonoyim BM, Frouin A, Li S, Ramakrishnan S, Merry KM, Shi Q, Rosenthal A, Barres BA (2016). Complement and microglia mediate early synapse loss in Alzheimer mouse models. Science.

[19] Rajendran L, Paolicelli RC (2018). Microglia-Mediated Synapse Loss in Alzheimer's Disease. J Neurosci.

[20] Feng B, Freitas AE, Gorodetski L, Wang J, Tian R, Lee YR, et al. Planar cell polarity signaling components are a direct target of beta-amyloid-associated degeneration of glutamatergic synapses. Sci Adv. 2021;7(eabh2307). 10.1126/sciadv.abh2307 PMC8373119 34407949

[21] Thakar S, Wang L, Yu T, Ye M, Onishi K, Scott J, Qi J, Fernandes C, Han X, Yates JR (2017). Evidence for opposing roles of Celsr3 and Vangl2 in glutamatergic synapse formation. Proc Natl Acad Sci U S A.

[22] Lambert JC, Heath S, Even G, Campion D, Sleegers K, Hiltunen M, Combarros O, Zelenika D, Bullido MJ, Tavernier B (2009). Genome-wide association study identifies variants at CLU and CR1 associated with Alzheimer's disease. Nat Genet.

[23] Harold D, Abraham R, Hollingworth P, Sims R, Gerrish A, Hamshere ML, Pahwa JS, Moskvina V, Dowzell K, Williams A (2009). Genome-wide association study identifies variants at CLU and PICALM associated with Alzheimer's disease. Nat Genet.

[24] Jansen IE, Savage JE, Watanabe K, Bryois J, Williams DM, Steinberg S, Sealock J, Karlsson IK, Hagg S, Athanasiu L (2019). Genome-wide meta-analysis identifies new loci and functional pathways influencing Alzheimer's disease risk. Nat Genet.

[25] Krasemann S, Madore C, Cialic R, Baufeld C, Calcagno N, El Fatimy R, Beckers L, O'Loughlin E, Xu Y, Fanek Z (2017). The TREM2-APOE Pathway Drives the Transcriptional Phenotype of Dysfunctional Microglia in Neurodegenerative Diseases. Immunity.

[26] Keren-Shaul H, Spinrad A, Weiner A, Matcovitch-Natan O, Dvir-Szternfeld R, Ulland TK, David E, Baruch K, Lara-Astaiso D, Toth B (2017). A Unique Microglia Type Associated with Restricting Development of Alzheimer's Disease. Cell.

[27] Douvaras P, Sun B, Wang M, Kruglikov I, Lallos G, Zimmer M, Terrenoire C, Zhang B, Gandy S, Schadt E (2017). Directed Differentiation of Human Pluripotent Stem Cells to Microglia. Stem Cell Reports.

[28] Zhang B, Gaiteri C, Bodea LG, Wang Z, McElwee J, Podtelezhnikov AA, Zhang C, Xie T, Tran L, Dobrin R (2013). Integrated systems approach identifies genetic nodes and networks in late-onset Alzheimer's disease. Cell.

[29] Hickman SE, Kingery ND, Ohsumi TK, Borowsky ML, Wang LC, Means TK, El Khoury J (2013). The microglial sensome revealed by direct RNA sequencing. Nat Neurosci.

[30] Audrain M, Haure-Mirande JV, Mleczko J, Wang M, Griffin JK, St George-Hyslop PH, Fraser P, Zhang B, Gandy S, Ehrlich ME (2021). Reactive or transgenic increase in microglial TYROBP reveals a TREM2-independent TYROBP-APOE link in wild-type and Alzheimer's-related mice. Alzheimers Dement.

[31] Audrain M, Haure-Mirande JV, Wang M, Kim SH, Fanutza T, Chakrabarty P, Fraser P, St George-Hyslop PH, Golde TE, Blitzer RD (2019). Integrative approach to sporadic Alzheimer's disease: deficiency of TYROBP in a tauopathy mouse model reduces C1q and normalizes clinical phenotype while increasing spread and state of phosphorylation of tau. Mol Psychiatry.

[32] Haure-Mirande JV, Wang M, Audrain M, Fanutza T, Kim SH, Heja S, Readhead B, Dudley JT, Blitzer RD, Schadt EE (2019). Integrative approach to sporadic Alzheimer's disease: deficiency of TYROBP in cerebral Abeta amyloidosis mouse normalizes clinical phenotype and complement subnetwork molecular pathology without reducing Abeta burden. Mol Psychiatry.

[33] Haure-Mirande JV, Audrain M, Fanutza T, Kim SH, Klein WL, Glabe C, Readhead B, Dudley JT, Blitzer RD, Wang M (2017). Deficiency of TYROBP, an adapter protein for TREM2 and CR3 receptors, is neuroprotective in a mouse model of early Alzheimer's pathology. Acta Neuropathol.

[34] Fowler AJ, Hebron M, Missner AA, Wang R, Gao X, Kurd-Misto BT, Liu X, Moussa CE (2019). Multikinase Abl/DDR/Src Inhibition Produces Optimal Effects for Tyrosine Kinase Inhibition in Neurodegeneration. Drugs R D.

[35] Chakrabarty P, Li A, Ceballos-Diaz C, Eddy JA, Funk CC, Moore B, DiNunno N, Rosario AM, Cruz PE, Verbeeck C (2015). IL-10 alters immunoproteostasis in APP mice, increasing plaque burden and worsening cognitive behavior. Neuron.

[36] Heneka MT, Sastre M, Dumitrescu-Ozimek L, Dewachter I, Walter J, Klockgether T, Van Leuven F (2005). Focal glial activation coincides with increased BACE1 activation and precedes amyloid plaque deposition in APP [V717I] transgenic mice. J Neuroinflammation.

[37] Griffin WS, Sheng JG, Royston MC, Gentleman SM, McKenzie JE, Graham DI, Roberts GW, Mrak RE (1998). Glial-neuronal interactions in Alzheimer's disease: the potential role of a 'cytokine cycle' in disease progression. Brain Pathol.

[38] Ringheim GE, Szczepanik AM, Petko W, Burgher KL, Zhu SZ, Chao CC (1998). Enhancement of beta-amyloid precursor protein transcription and expression by the soluble interleukin-6 receptor/interleukin-6 complex. Brain Res Mol Brain Res.

[39] Olcese L, Cambiaggi A, Semenzato G, Bottino C, Moretta A, Vivier E (1997). Human killer cell activatory receptors for MHC class I molecules are included in a multimeric complex expressed by natural killer cells. J Immunol.

[40] Lanier LL, Corliss BC, Wu J, Leong C, Phillips JH (1998). Immunoreceptor DAP12 bearing a tyrosine-based activation motif is involved in activating NK cells. Nature.

[41] Smith KM, Wu J, Bakker AB, Phillips JH, Lanier LL (1998). Ly-49D and Ly-49H associate with mouse DAP12 and form activating receptors. J Immunol.

[42] Ziegenfuss JS, Biswas R, Avery MA, Hong K, Sheehan AE, Yeung YG, Stanley ER, Freeman MR (2008). Draper-dependent glial phagocytic activity is mediated by Src and Syk family kinase signalling. Nature.

[43] Call ME, Wucherpfennig KW, Chou JJ (2010). The structural basis for intramembrane assembly of an activating immunoreceptor complex. Nat Immunol.

[44] Feng J, Call ME, Wucherpfennig KW (2006). The assembly of diverse immune receptors is focused on a polar membrane-embedded interaction site. PLoS Biol.

[45] Hamerman JA, Jarjoura JR, Humphrey MB, Nakamura MC, Seaman WE, Lanier LL (2006). Cutting edge: inhibition of TLR and FcR responses in macrophages by triggering receptor expressed on myeloid cells (TREM)-2 and DAP12. J Immunol.

[46] Hamerman JA, Tchao NK, Lowell CA, Lanier LL (2005). Enhanced Toll-like receptor responses in the absence of signaling adaptor DAP12. Nat Immunol.

[47] Turnbull IR, Gilfillan S, Cella M, Aoshi T, Miller M, Piccio L, Hernandez M, Colonna M (2006). Cutting edge: TREM-2 attenuates macrophage activation. J Immunol.

[48] Fuchs A, Cella M, Kondo T, Colonna M (2005). Paradoxic inhibition of human natural interferon-producing cells by the activating receptor NKp44. Blood.

[49] Turnbull IR, Colonna M (2007). Activating and inhibitory functions of DAP12. Nat Rev Immunol.

[50] Lanier LL (2009). DAP10- and DAP12-associated receptors in innate immunity. Immunol Rev.

[51] Angata T (2020). Siglecs that Associate with DAP12. Adv Exp Med Biol.

[52] Dietrich J, Cella M, Seiffert M, Buhring HJ, Colonna M (2000). Cutting edge: signal-regulatory protein beta 1 is a DAP12-associated activating receptor expressed in myeloid cells. J Immunol.

[53] Tomasello E, Cant C, Buhring HJ, Vely F, Andre P, Seiffert M, Ullrich A, Vivier E (2000). Association of signal-regulatory proteins beta with KARAP/DAP-12. Eur J Immunol.

[54] Hayashi A, Ohnishi H, Okazawa H, Nakazawa S, Ikeda H, Motegi S, Aoki N, Kimura S, Mikuni M, Matozaki T (2004). Positive regulation of phagocytosis by SIRPbeta and its signaling mechanism in macrophages. J Biol Chem.

[55] Gaikwad S, Larionov S, Wang Y, Dannenberg H, Matozaki T, Monsonego A, Thal DR, Neumann H (2009). Signal regulatory protein-beta1: a microglial modulator of phagocytosis in Alzheimer's disease. Am J Pathol.

[56] Bouchon A, Dietrich J, Colonna M (2000). Cutting edge: inflammatory responses can be triggered by TREM-1, a novel receptor expressed on neutrophils and monocytes. J Immunol.

[57] Fu L, Han L, Xie C, Li W, Lin L, Pan S, Zhou Y, Li Z, Jin M, Zhang A (2017). Identification of Extracellular Actin As a Ligand for Triggering Receptor Expressed on Myeloid Cells-1 Signaling. Front Immunol.

[58] Read CB, Kuijper JL, Hjorth SA, Heipel MD, Tang X, Fleetwood AJ, Dantzler JL, Grell SN, Kastrup J, Wang C (2015). Cutting Edge: identification of neutrophil PGLYRP1 as a ligand for TREM-1. J Immunol.

[59] El Mezayen R, El Gazzar M, Seeds MC, McCall CE, Dreskin SC, Nicolls MR (2007). Endogenous signals released from necrotic cells augment inflammatory responses to bacterial endotoxin. Immunol Lett.

[60] Arts RJ, Joosten LA, Dinarello CA, Kullberg BJ, van der Meer JW, Netea MG (2011). TREM-1 interaction with the LPS/TLR4 receptor complex. Eur Cytokine Netw.

[61] Bouchon A, Facchetti F, Weigand MA, Colonna M (2001). TREM-1 amplifies inflammation and is a crucial mediator of septic shock. Nature.

[62] Lagler H, Sharif O, Haslinger I, Matt U, Stich K, Furtner T, Doninger B, Schmid K, Gattringer R, de Vos AF, Knapp S (2009). TREM-1 activation alters the dynamics of pulmonary IRAK-M expression in vivo and improves host defense during pneumococcal pneumonia. J Immunol.

[63] Atagi Y, Liu CC, Painter MM, Chen XF, Verbeeck C, Zheng H, Li X, Rademakers R, Kang SS, Xu H (2015). Apolipoprotein E Is a Ligand for Triggering Receptor Expressed on Myeloid Cells 2 (TREM2). J Biol Chem.

[64] Bailey CC, DeVaux LB, Farzan M (2015). The Triggering Receptor Expressed on Myeloid Cells 2 Binds Apolipoprotein E. J Biol Chem.

[65] Bouchon A, Hernandez-Munain C, Cella M, Colonna M (2001). A DAP12-mediated pathway regulates expression of CC chemokine receptor 7 and maturation of human dendritic cells. J Exp Med.

[66] Daws MR, Sullam PM, Niemi EC, Chen TT, Tchao NK, Seaman WE (2003). Pattern recognition by TREM-2: binding of anionic ligands. J Immunol.

[67] Pottier C, Wallon D, Rousseau S, Rovelet-Lecrux A, Richard AC, Rollin-Sillaire A, Frebourg T, Campion D, Hannequin D (2013). TREM2 R47H variant as a risk factor for early-onset Alzheimer's disease. J Alzheimers Dis.

[68] Soragna D, Papi L, Ratti MT, Sestini R, Tupler R, Montalbetti L (2003). An Italian family affected by Nasu-Hakola disease with a novel genetic mutation in the TREM2 gene. J Neurol Neurosurg Psychiatry.

[69] Borroni B, Ferrari F, Galimberti D, Nacmias B, Barone C, Bagnoli S, Fenoglio C, Piaceri I, Archetti S, Bonvicini C (2014). Heterozygous TREM2 mutations in frontotemporal dementia. Neurobiol Aging.

[70] Chung DH, Seaman WE, Daws MR (2002). Characterization of TREM-3, an activating receptor on mouse macrophages: definition of a family of single Ig domain receptors on mouse chromosome 17. Eur J Immunol.

[71] Martinez-Barriocanal A, Sayos J (2006). Molecular and functional characterization of CD300b, a new activating immunoglobulin receptor able to transduce signals through two different pathways. J Immunol.

[72] Bakker AB, Baker E, Sutherland GR, Phillips JH, Lanier LL (1999). Myeloid DAP12-associating lectin (MDL)-1 is a cell surface receptor involved in the activation of myeloid cells. Proc Natl Acad Sci U S A.

[73] Teng O, Chen ST, Hsu TL, Sia SF, Cole S, Valkenburg SA, Hsu TY, Zheng JT, Tu W, Bruzzone R (2017). CLEC5A-Mediated Enhancement of the Inflammatory Response in Myeloid Cells Contributes to Influenza Virus Pathogenicity In Vivo. J Virol.

[74] Sung PS, Hsieh SL (2019). CLEC2 and CLEC5A: Pathogenic Host Factors in Acute Viral Infections. Front Immunol.

[75] Wortham BW, Eppert BL, Flury JL, Garcia SM, Donica WR, Osterburg A, Joyce-Shaikh B, Cua DJ, Borchers MT (2016). Cutting Edge: CLEC5A Mediates Macrophage Function and Chronic Obstructive Pulmonary Disease Pathologies. J Immunol.

[76] Angata T, Hayakawa T, Yamanaka M, Varki A, Nakamura M (2006). Discovery of Siglec-14, a novel sialic acid receptor undergoing concerted evolution with Siglec-5 in primates. FASEB J.

[77] Yamanaka M, Kato Y, Angata T, Narimatsu H (2009). Deletion polymorphism of SIGLEC14 and its functional implications. Glycobiology.

[78] Angata T, Tabuchi Y, Nakamura K, Nakamura M (2007). Siglec-15: an immune system Siglec conserved throughout vertebrate evolution. Glycobiology.

[79] Chang L, Chen YJ, Fan CY, Tang CJ, Chen YH, Low PY, Ventura A, Lin CC, Chen YJ, Angata T (2017). Identification of Siglec Ligands Using a Proximity Labeling Method. J Proteome Res.

[80] Hiruma Y, Tsuda E, Maeda N, Okada A, Kabasawa N, Miyamoto M, Hattori H, Fukuda C (2013). Impaired osteoclast differentiation and function and mild osteopetrosis development in Siglec-15-deficient mice. Bone.

[81] Cao H, Lakner U, de Bono B, Traherne JA, Trowsdale J, Barrow AD (2008). SIGLEC16 encodes a DAP12-associated receptor expressed in macrophages that evolved from its inhibitory counterpart SIGLEC11 and has functional and non-functional alleles in humans. Eur J Immunol.

[82] Blasius AL, Cella M, Maldonado J, Takai T, Colonna M (2006). Siglec-H is an IPC-specific receptor that modulates type I IFN secretion through DAP12. Blood.

[83] Konishi H, Kobayashi M, Kunisawa T, Imai K, Sayo A, Malissen B, Crocker PR, Sato K, Kiyama H (2017). Siglec-H is a microglia-specific marker that discriminates microglia from CNS-associated macrophages and CNS-infiltrating monocytes. Glia.

[84] Ford JW, McVicar DW (2009). TREM and TREM-like receptors in inflammation and disease. Curr Opin Immunol.

[85] Mousseau DD, Banville D, L'Abbe D, Bouchard P, Shen SH (2000). PILRalpha, a novel immunoreceptor tyrosine-based inhibitory motif-bearing protein, recruits SHP-1 upon tyrosine phosphorylation and is paired with the truncated counterpart PILRbeta. J Biol Chem.

[86] Shiratori I, Ogasawara K, Saito T, Lanier LL, Arase H (2004). Activation of natural killer cells and dendritic cells upon recognition of a novel CD99-like ligand by paired immunoglobulin-like type 2 receptor. J Exp Med.

[87] Wang J, Shiratori I, Satoh T, Lanier LL, Arase H (2008). An essential role of sialylated O-linked sugar chains in the recognition of mouse CD99 by paired Ig-like type 2 receptor (PILR). J Immunol.

[88] Tabata S, Kuroki K, Wang J, Kajikawa M, Shiratori I, Kohda D, Arase H, Maenaka K (2008). Biophysical characterization of O-glycosylated CD99 recognition by paired Ig-like type 2 receptors. J Biol Chem.

[89] Lambert JC, Ibrahim-Verbaas CA, Harold D, Naj AC, Sims R, Bellenguez C, DeStafano AL, Bis JC, Beecham GW, Grenier-Boley B (2013). Meta-analysis of 74,046 individuals identifies 11 new susceptibility loci for Alzheimer's disease. Nat Genet.

[90] Walker DG, Whetzel AM, Serrano G, Sue LI, Beach TG, Lue LF (2015). Association of CD33 polymorphism rs3865444 with Alzheimer's disease pathology and CD33 expression in human cerebral cortex. Neurobiol Aging.

[91] Griciuc A, Serrano-Pozo A, Parrado AR, Lesinski AN, Asselin CN, Mullin K, Hooli B, Choi SH, Hyman BT, Tanzi RE (2013). Alzheimer's disease risk gene CD33 inhibits microglial uptake of amyloid beta. Neuron.

[92] Yamanishi Y, Kitaura J, Izawa K, Matsuoka T, Oki T, Lu Y, Shibata F, Yamazaki S, Kumagai H, Nakajima H (2008). Analysis of mouse LMIR5/CLM-7 as an activating receptor: differential regulation of LMIR5/CLM-7 in mouse versus human cells. Blood.

[93] Aguilar H, Alvarez-Errico D, Garcia-Montero AC, Orfao A, Sayos J, Lopez-Botet M (2004). Molecular characterization of a novel immune receptor restricted to the monocytic lineage. J Immunol.

[94] Luo K, Zhang W, Sui L, Li N, Zhang M, Ma X, Zhang L, Cao X (2001). DIgR1, a novel membrane receptor of the immunoglobulin gene superfamily, is preferentially expressed by antigen-presenting cells. Biochem Biophys Res Commun.

[95] Kumagai H, Oki T, Tamitsu K, Feng SZ, Ono M, Nakajima H, Bao YC, Kawakami Y, Nagayoshi K, Copeland NG (2003). Identification and characterization of a new pair of immunoglobulin-like receptors LMIR1 and 2 derived from murine bone marrow-derived mast cells. Biochem Biophys Res Commun.

[96] Chung DH, Humphrey MB, Nakamura MC, Ginzinger DG, Seaman WE, Daws MR (2003). CMRF-35-like molecule-1, a novel mouse myeloid receptor, can inhibit osteoclast formation. J Immunol.

[97] Jonsson T, Stefansson H, Steinberg S, Jonsdottir I, Jonsson PV, Snaedal J, Bjornsson S, Huttenlocher J, Levey AI, Lah JJ (2013). Variant of TREM2 associated with the risk of Alzheimer's disease. N Engl J Med.

[98] Guerreiro R, Wojtas A, Bras J, Carrasquillo M, Rogaeva E, Majounie E, Cruchaga C, Sassi C, Kauwe JS, Younkin S (2013). TREM2 variants in Alzheimer's disease. N Engl J Med.

[99] Yeh FL, Wang Y, Tom I, Gonzalez LC, Sheng M (2016). TREM2 Binds to Apolipoproteins, Including APOE and CLU/APOJ, and Thereby Facilitates Uptake of Amyloid-Beta by Microglia. Neuron.

[100] Zhong L, Chen XF, Wang T, Wang Z, Liao C, Wang Z, Huang R, Wang D, Li X, Wu L (2017). Soluble TREM2 induces inflammatory responses and enhances microglial survival. J Exp Med.

[101] Lessard CB, Malnik SL, Zhou Y, Ladd TB, Cruz PE, Ran Y, Mahan TE, Chakrabaty P, Holtzman DM, Ulrich JD (2018). High-affinity interactions and signal transduction between Abeta oligomers and TREM2. EMBO Mol Med.

[102] Vilalta A, Zhou Y, Sevalle J, Griffin JK, Satoh K, Allendorf DH, De S, Puigdellivol M, Bruzas A, Burguillos MA (2021). Wild-type sTREM2 blocks Abeta aggregation and neurotoxicity, but the Alzheimer's R47H mutant increases Abeta aggregation. J Biol Chem.

[103] Suarez-Calvet M, Morenas-Rodriguez E, Kleinberger G, Schlepckow K, Araque Caballero MA, Franzmeier N, Capell A, Fellerer K, Nuscher B, Eren E (2019). Early increase of CSF sTREM2 in Alzheimer's disease is associated with tau related-neurodegeneration but not with amyloid-beta pathology. Mol Neurodegener.

[104] Yang J, Fu Z, Zhang X, Xiong M, Meng L, Zhang Z (2020). TREM2 ectodomain and its soluble form in Alzheimer's disease. J Neuroinflammation.

[105] Gisslen M, Heslegrave A, Veleva E, Yilmaz A, Andersson LM, Hagberg L, Spudich S, Fuchs D, Price RW, Zetterberg H (2019). CSF concentrations of soluble TREM2 as a marker of microglial activation in HIV-1 infection. Neurol Neuroimmunol Neuroinflamm.

[106] Daws MR, Lanier LL, Seaman WE, Ryan JC (2001). Cloning and characterization of a novel mouse myeloid DAP12-associated receptor family. Eur J Immunol.

[107] Zhang J, Raper A, Sugita N, Hingorani R, Salio M, Palmowski MJ, Cerundolo V, Crocker PR (2006). Characterization of Siglec-H as a novel endocytic receptor expressed on murine plasmacytoid dendritic cell precursors. Blood.

[108] Blasius A, Vermi W, Krug A, Facchetti F, Cella M, Colonna M (2004). A cell-surface molecule selectively expressed on murine natural interferon-producing cells that blocks secretion of interferon-alpha. Blood.

[109] Griciuc A, Patel S, Federico AN, Choi SH, Innes BJ, Oram MK, Cereghetti G, McGinty D, Anselmo A, Sadreyev RI (2019). TREM2 Acts Downstream of CD33 in Modulating Microglial Pathology in Alzheimer's Disease. Neuron.

[110] Angata T, Reynolds SA, Powell LD, Hedrick SM, Varki A, Brinkman-Van der Linden EC (2003). CD33/Siglec-3 binding specificity, expression pattern, and consequences of gene deletion in mice. Mol Cell Biol.

[111] Taylor VC, Buckley CD, Douglas M, Cody AJ, Simmons DL, Freeman SD (1999). The myeloid-specific sialic acid-binding receptor, CD33, associates with the protein-tyrosine phosphatases, SHP-1 and SHP-2. J Biol Chem.

[112] Ulyanova T, Blasioli J, Woodford-Thomas TA, Thomas ML (1999). The sialoadhesin CD33 is a myeloid-specific inhibitory receptor. Eur J Immunol.

[113] Paul SP, Taylor LS, Stansbury EK, McVicar DW (2000). Myeloid specific human CD33 is an inhibitory receptor with differential ITIM function in recruiting the phosphatases SHP-1 and SHP-2. Blood.

[114] Chen W-T, Lu A, Craessaerts K, Pavie B, Sala Frigerio C, Corthout N, Qian X, Laláková J, Kühnemund M, Voytyuk I (2020). Spatial Transcriptomics and In Situ Sequencing to Study Alzheimer’s Disease. Cell.

[115] Pottier C, Ravenscroft TA, Brown PH, Finch NA, Baker M, Parsons M, Asmann YW, Ren Y, Christopher E, Levitch D (2016). TYROBP genetic variants in early-onset Alzheimer's disease. Neurobiol Aging.

[116] Paloneva J, Kestila M, Wu J, Salminen A, Bohling T, Ruotsalainen V, Hakola P, Bakker AB, Phillips JH, Pekkarinen P (2000). Loss-of-function mutations in TYROBP (DAP12) result in a presenile dementia with bone cysts. Nat Genet.

[117] Masuda T, Tsuda M, Yoshinaga R, Tozaki-Saitoh H, Ozato K, Tamura T, Inoue K (2012). IRF8 is a critical transcription factor for transforming microglia into a reactive phenotype. Cell Rep.

[118] d'Errico P, Ziegler-Waldkirch S, Aires V, Hoffmann P, Mezo C, Erny D, Monasor LS, Liebscher S, Ravi VM, Joseph K (2022). Microglia contribute to the propagation of Abeta into unaffected brain tissue. Nat Neurosci.

[119] Kierdorf K, Erny D, Goldmann T, Sander V, Schulz C, Perdiguero EG, Wieghofer P, Heinrich A, Riemke P, Holscher C (2013). Microglia emerge from erythromyeloid precursors via Pu.1- and Irf8-dependent pathways. Nat Neurosci.

[120] Fonseca MI, Chu SH, Hernandez MX, Fang MJ, Modarresi L, Selvan P, MacGregor GR, Tenner AJ (2017). Cell-specific deletion of C1qa identifies microglia as the dominant source of C1q in mouse brain. J Neuroinflammation.

[121] Jiang J, Ding Y, Wu M, Lyu X, Wang H, Chen Y, Wang H, Teng L (2020). Identification of TYROBP and C1QB as Two Novel Key Genes With Prognostic Value in Gastric Cancer by Network Analysis. Front Oncol.

[122] Dejanovic B, Huntley MA, De Maziere A, Meilandt WJ, Wu T, Srinivasan K, Jiang Z, Gandham V, Friedman BA, Ngu H (2018). Changes in the Synaptic Proteome in Tauopathy and Rescue of Tau-Induced Synapse Loss by C1q Antibodies. Neuron.

[123] Otero K, Turnbull IR, Poliani PL, Vermi W, Cerutti E, Aoshi T, Tassi I, Takai T, Stanley SL, Miller M (2009). Macrophage colony-stimulating factor induces the proliferation and survival of macrophages via a pathway involving DAP12 and beta-catenin. Nat Immunol.

[124] Mocsai A, Abram CL, Jakus Z, Hu Y, Lanier LL, Lowell CA (2006). Integrin signaling in neutrophils and macrophages uses adaptors containing immunoreceptor tyrosine-based activation motifs. Nat Immunol.

[125] Wakselman S, Bechade C, Roumier A, Bernard D, Triller A, Bessis A (2008). Developmental neuronal death in hippocampus requires the microglial CD11b integrin and DAP12 immunoreceptor. J Neurosci.

[126] Shi Q, Chowdhury S, Ma R, Le KX, Hong S, Caldarone BJ, et al. Complement C3 deficiency protects against neurodegeneration in aged plaque-rich APP/PS1 mice. Sci Transl Med. 2017;9. 10.1126/scitranslmed.aaf6295 PMC6936623 28566429

[127] Huang Y, Happonen KE, Burrola PG, O'Connor C, Hah N, Huang L, Nimmerjahn A, Lemke G (2021). Microglia use TAM receptors to detect and engulf amyloid beta plaques. Nat Immunol.

[128] Butovsky O, Jedrychowski MP, Cialic R, Krasemann S, Murugaiyan G, Fanek Z, Greco DJ, Wu PM, Doykan CE, Kiner O (2015). Targeting miR-155 restores abnormal microglia and attenuates disease in SOD1 mice. Ann Neurol.

[129] Readhead B, Haure-Mirande JV, Mastroeni D, Audrain M, Fanutza T, Kim SH, Blitzer RD, Gandy S, Dudley JT, Ehrlich ME (2020). miR155 regulation of behavior, neuropathology, and cortical transcriptomics in Alzheimer's disease. Acta Neuropathol.

[130] Nasu T, Tsukahara Y, Terayama K (1973). A lipid metabolic disease-"membranous lipodystrophy"-an autopsy case demonstrating numerous peculiar membrane-structures composed of compound lipid in bone and bone marrow and various adipose tissues. Acta Pathol Jpn.

[131] Hakola HP, Iivanainen M (1973). A new hereditary disease with progressive dementia and polycystic osteodysplasia: neuroradiological analysis of seven cases. Neuroradiology.

[132] Ghezzi L, Carandini T, Arighi A, Fenoglio C, Arcaro M, De Riz M, Pietroboni AM, Fumagalli GG, Basilico P, Calvi A (2017). Evidence of CNS beta-amyloid deposition in Nasu-Hakola disease due to the TREM2 Q33X mutation. Neurology.

[133] Satoh JI, Kino Y, Yanaizu M, Saito Y (2018). Alzheimer's disease pathology in Nasu-Hakola disease brains. Intractable Rare Dis Res.

[134] McVicar DW, Trinchieri G (2009). CSF-1R, DAP12 and beta-catenin: a menage a trois. Nat Immunol.

[135] Rademakers R, Baker M, Nicholson AM, Rutherford NJ, Finch N, Soto-Ortolaza A, Lash J, Wider C, Wojtas A, DeJesus-Hernandez M (2011). Mutations in the colony stimulating factor 1 receptor (CSF1R) gene cause hereditary diffuse leukoencephalopathy with spheroids. Nat Genet.

[136] Golde TE, DeKosky ST, Galasko D (2018). Alzheimer's disease: The right drug, the right time. Science.

[137] Parhizkar S, Arzberger T, Brendel M, Kleinberger G, Deussing M, Focke C, Nuscher B, Xiong M, Ghasemigharagoz A, Katzmarski N (2019). Loss of TREM2 function increases amyloid seeding but reduces plaque-associated ApoE. Nat Neurosci.

[138] Schoch KM, Ezerskiy LA, Morhaus MM, Bannon RN, Sauerbeck AD, Shabsovich M, et al. Acute Trem2 reduction triggers increased microglial phagocytosis, slowing amyloid deposition in mice. Proc Natl Acad Sci U S A. 2021;118. 10.1073/pnas.2100356118 PMC8271763 34187891

[139] Ivashkiv LB (2009). Cross-regulation of signaling by ITAM-associated receptors. Nat Immunol.

[140] Ferrara SJ, Chaudhary P, DeBell MJ, Marracci G, Miller H, Calkins E, Pocius E, Napier BA, Emery B, Bourdette D, Scanlan TS (2022). TREM2 is thyroid hormone regulated making the TREM2 pathway druggable with ligands for thyroid hormone receptor. Cell Chem Biol.

